# The Rigid Double Harmonic Oscillator Model of DNA Base Pairs and the Wignerian Approach for the Transition Rate of Single-Proton Transfer within the Fermi Golden Rule

**DOI:** 10.1021/acs.jpcb.5c08297

**Published:** 2026-02-19

**Authors:** M. Wleklińska, B. J. Spisak

**Affiliations:** AGH University of Krakow, Faculty of Physics and Applied Computer Science, al. A. Mickiewicza 30, 30-059 Krakow, Poland

## Abstract

An exactly solvable quantum-mechanical model of DNA bases is presented. The proposed model is based on the harmonic approximation of the potential energy of the canonical and tautomeric forms for the bases in question. The transition rate of single-proton transfer in the base pairs adenine–thymine and guanine–cytosine is determined within this model using the Fermi Golden Rule. The transition rates derived from the application of the ‘bare’ Fermi Golden Rule can be approximated by the Fano line curves. In turn, the application of the thermally averaged Fermi Golden Rule leads to an exponential increase in transition rates across the biologically relevant temperature range for the base pairs under consideration. The kinetic isotope effect for the base pairs at room temperature is also estimated. All calculations are consistently carried out in a phase-space representation using the Wigner distribution function.

## Graphical abstract

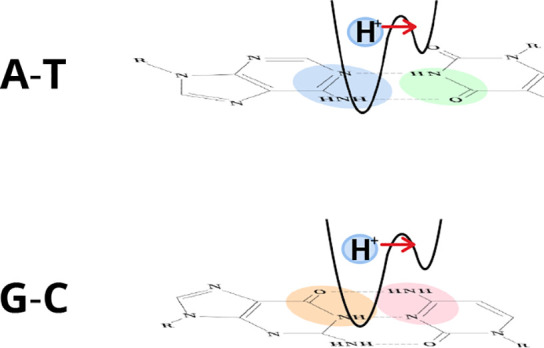

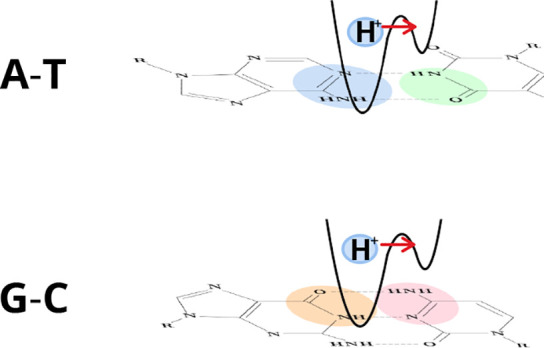

## Introduction

The discovery of the DNA structure by Watson and Crick^1^ with Franklin^2^ elucidated that this long polymer in the form of the double helix is responsible for the characteristics and biological functions of an organism. According to this finding, each DNA strand consists of the sugar–phosphate backbone, which supports nitrogenous bases. Four different DNA bases, adenine, thymine, cytosine and guanine, are observed. Because of the complementary base pairing, these bases form stable pairs: adenine–thymine (A–T) and guanine–cytosine (G–C). Moreover, such stable pairings of DNA bases in the double helix is essential for replication and transcription. However, it is worth noting that their structures can be altered via double proton transfer between the bases.^1,3^ As a result, tautomeric forms of the bases are generated due to proton hopping within the bases, which slightly alters the bonding arrangement within the molecule. These forms mimic the geometry of the correct pairings, making them undetectable to DNA polymerases and repair. Consequently, they can cause incorrect pairing during DNA replication, which can then lead to point mutations. This means these seemingly unimportant proton movements cause structural changes in the DNA bases, as shown in Figure 1. It is crucial to note that a single proton transfer is rather a rare event, but it cannot be overlooked because it typically triggers the second transfer.^4^ This observation leads to a a reasonably key question: How can the transition rate for a single proton between A–T and G–C base pairs be estimated? Motivated by this question, we developed a quantitative analysis of this process, assuming that the proton transfer can be regarded as the tunnelling process, according to original Löwdin’s idea.^3^ It seems to be that our approach is nothing unusual because applying quantum description to quantitative investigations of various characteristics of biological structures is becoming increasingly successful, and consequently, it has given rise to the development of a new field of research, namely quantum biology.^5–8^

**Figure 1:**
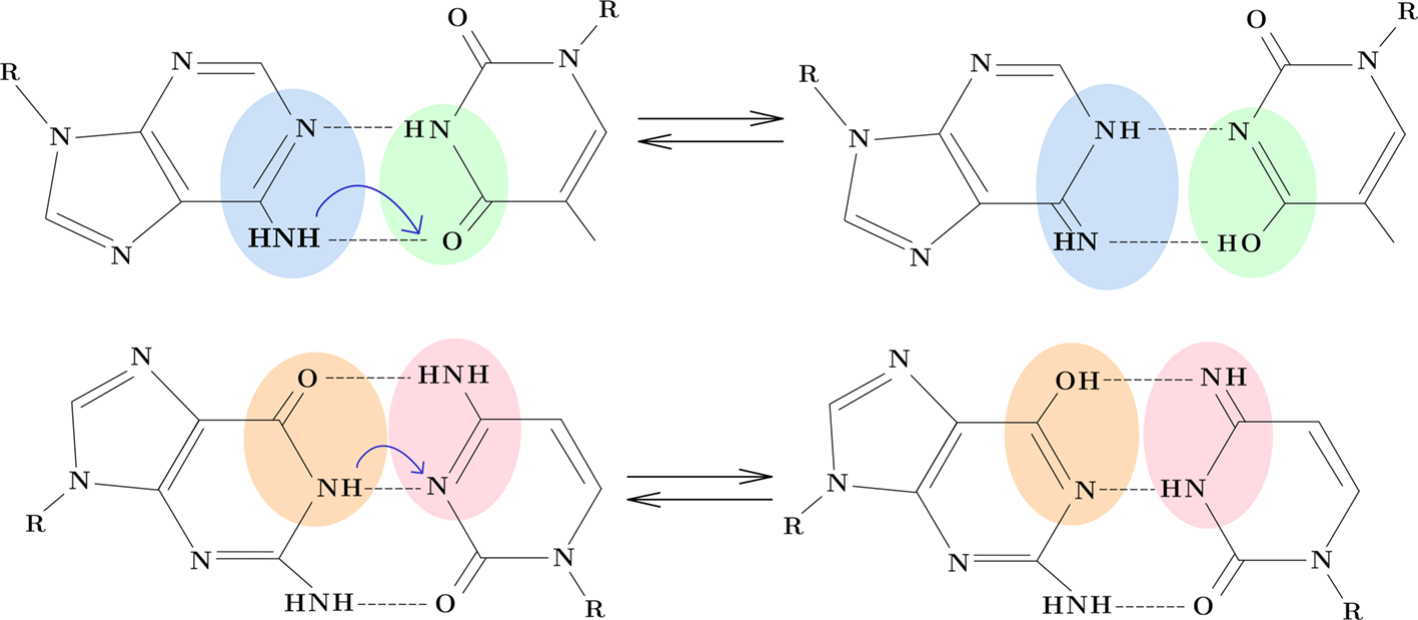
Schematics of double proton transfer due to tautomeric changes in DNA bases (upper figure: A–T; lower figure: G–C).

The tautomerisation of DNA base pairs has been studied comprehensively,^3,9–16^ although the exact mechanism and the external factors influencing it remain uncertain. Most studies have focused on the dynamical aspects of tautomerisation,^9,16,17^ modeling the time evolution of the system and the time-dependent changes in tautomerisation probability, often incorporating external factors such as temperature or applied electric fields.

In this study, we present an exactly solvable quantum-mechanical model of single-proton transfer between DNA base pairs. This model is based on the harmonic approximation of the potential energy of the canonical and tautomeric forms, which are separated by the parabolic barrier. The transition rate between A–T and G–C base pairs is determined by using the Fermi Golden Rule (FGR). For this purpose, we consistently applied the phase-space representation of the quantum theory using the Wigner distribution function. The choice of this representation is partly dictated by the possibility of a relatively simple generalization of the proposed model to the dynamical case,^12,16,18,19^ including the interaction with the environment,^13–15^ or generalized to the case of two-proton transfer within the mean-field approximation, as we qualitatively discuss.

The rest of the paper is organized as follows. The second section provides a theoretical framework for the conducted studies, including the harmonic model of DNA for A–T and G–C base pairs, the basic concepts related to the time-independent Moyal equation for the WDF, and its solution. In the third section, we present the main results of our studies on single-proton transfer, based on the application of the FGR, and discuss them. In the last section, we summarize the key findings and conclusions. The Supporting Information is an integral part of these studies and contains technical results directly related to the theoretical and numerical aspects presented herein.

## Theoretical Framework

The key concept for describing the state of the quantum system within the phase-space representation is the Wigner distribution function (WDF). This function is not a properly defined probability distribution function because it can take negative values in some regions of the phase space. Hence, it is customarily treated as the quasi-distribution function because the rest of its properties are consistent with the classical definition of the probability distribution function, especially since it is real, normalized in a sense $L^{1}(dxdp,R^{2})$ – norm, possess well-defined marginals distribution. Furthermore, it can be used to determine expectation values of dynamic variables using the phase-space integrals in the same manner as in statistical mechanics. It is also noteworthy that, the WDF can be regarded as a special case of the Weyl symbol of the Hermitian operator Ω̂ = Ω­(*x̂*, *p̂*) associated with the classical dynamical variable Ω, and which is given by the formula

$$\Omega (x,p)=\int dX\left\langle x-\frac{1}{2}X|\hat{\Omega }|x+\frac{1}{2}X\right\rangle e^{-i/\hbar pX}$$ 1

where the Dirac notation is used, and rest of the symbols have the usual meaning, i.e., *i* is the imaginary unit (*i* ^2^ = −1), and *ℏ* is the reduced Planck constant. Simply, for the WDF, the operator Ω̂ has the form ρ̂/(2π*ℏ*), where ρ̂ is the density operator. In the presented approach, the system in question is characterized by the Weyl symbol of the single-particle Hamiltonian of the following form: *H*_W_(*x*, *p*) = *p* ^2^/(2*m*) + *U*_W_(*x*), where *m* is the mass of the proton, *U*_W_(*x*) is the Weyl symbol of the potential energy, *p* and *x* are also the Weyl symbols of the momentum and position operators, respectively. The WDF corresponding to the stationary state of the system described by the above-mentioned Weyl symbol *H*_W_(*x*, *p*) can be determined by solving the eigenvalue equation (the time-independent Moyal equation) in the form^20^

$$\left[\frac{p^{2}}{2m}-i\frac{\hbar p}{2m}\partial_{x}-\frac{\hbar^{2}}{8m}\partial_{x}^{2}+\sum_{n=0}^{\infty }\frac{(i\hbar {)}^{n}}{2^{n}n!}\partial_{x}^{n}U_{W}(x)\partial_{p}^{n}\right]\varrho (x,p)=E\varrho (x,p)$$ 2

where *E* is the eigenvalue of the corresponding Weyl symbol *H*_W_(*x*, *p*). Let us note that this approach is restricted to the Weyl symbol of the potential energy, *U*(*x*), which is represented by a smooth function. It is also worth noting that the equation obtained in this manner is essentially of infinite order in momentum and position variables. However, this issue can be avoided by considering potential energy in polynomial form. As a result, only a finite number of derivative terms are involved, and the resulting equation remains finite order with respect to phase-space variables.^21^ The approach mentioned above can be applied to the quantitative discussion of the single-proton transfer between DNA base pairs, but the first step in this study requires specifying the potential energy of the proton. In accordance with the results presented in refs 9 and 18, the proton transfer potential energy profile is represented by the function in the form of the fourth-order polynomial for the A–T base pair, and the back-to-back double Morse for the G–C base pair. Despite the high utility of these kinds of potentials in numerical models, their usage in exactly solvable models is a rather complex task or even impossible to implement due to calculational difficulties. The natural question that arises from this statement concerns the possibility of formulating a simple, but not too simple, mathematical model for the proton transfer in the DNA base pairs that is exactly solvable. Thereby, it could serve as a minimal reference model necessary for interpreting the results obtained from more advanced calculations. Let us note that there is nothing unusual in such a strategy because such mathematical models in basic research are often encountered. For this purpose, we develop a model of the DNA base pairs based on the concept of two independent displacement harmonic oscillators (DHO), which are coupled by the inverted harmonic oscillator (IHO), whereby the IHO is treated as the perturbation. In other words, the proton potential energy profile, *U*(*x*), is expressed by the spline function in the form of a continuous combination of second-order polynomials, as follows

$$U(x)=\sum_{r=0}^{2}a_{r}^{\left(\tilde{F},l\right)}x^{r}=\{\begin{array}{ll} U^{\left(C,l\right)}(x), & x\in (-\infty ,x_{C})\\ U^{\left(B,l\right)}(x), & x\in (x_{C},x_{T})\\ U^{\left(T,l\right)}(x), & x\in (x_{T},+\infty ) \end{array}$$ 3

where the upper index *F̃* = C, T, B refers to canonical, tautomeric forms and barriers, respectively, and the second upper index *l* refers to specific base pair, i.e., A–T or G–C. In turn, *x*_*F̃*_ corresponds to the limiting points for each of the parts of this compose potential energy, and *a*_*r*_^(*F̃*,*l*)^ are real coefficients in quadratic polynomials for fixed pairs (*F̃*, *l*). The relationship between coefficients *a*_*r*_^(*F̃*,*l*)^ and the model parameter: ω^(*F̃*,*l*)^, *X* ^(*F̃*,*l*)^ and *C* ^(*F̃*,*l*)^ is presented in the Supporting Information. Then, we associate with each part of the potential energy ([Disp-formula eq3]) appropriate Weyl symbols *U*_W_^(*F*,*l*)^(*x*), according to the formula ([Disp-formula eq1]). In this notation, the Weyl symbols *U*_W_^(C,*l*)^(*x*) and *U*_W_^(T,*l*)^(*x*) have the form of the DHOs describing the canonical and tautomeric forms, respectively. Their general form is the following

$$U_{W}^{\left(F,l\right)}(x)=\frac{1}{2}m(\omega^{\left(F,l\right)}{)}^{2}(x-X^{\left(F,l\right)}{)}^{2}+C^{\left(F,l\right)}$$ 4

where *m* is the mass of the proton, ω^(*F*,*l*)^ is the angular frequency, *X* ^(*F*,*l*)^ is the position of the local energy minimum of the DHOs for the fixed pair (*F*, *l*), and *C* ^(*F*,*l*)^ is the placement of the local energy minimum of the DHO for the same pair (*F*, *l*). In turn, *U*_W_^(B,*l*)^(*x*) is the Weyl symbol of the potential energy of the IHO has the form

$$U_{W}^{\left(B,l\right)}(x)=-\frac{1}{2}m(\omega^{\left(B,l\right)}{)}^{2}(x-X^{\left(B,l\right)}{)}^{2}+C^{\left(B,l\right)}$$ 5

where *X* ^(*B*,*l*)^ is the position of the local energy maximum of the IHO for the fixed pair *l*, *C* ^(*B*,*l*)^ is the placement of the local energy maximum, and the angular frequency ω^(B,*l*)^.

Let us briefly summarize the main features of the proposed construction of the potential energy for the tunnelling proton. Each of the two independent DHOs is embedded in the local minima, corresponding to the canonical and tautomeric forms, and the IHO separates them. Its local maximum defines the height of the potential energy barrier for the proton tunnelling between the considered base pairs.

The advantage of this construction is that the proposed harmonic approximation of the energy profile of the tunnelling proton has an exact solution within the presented Wignerian approach. In fact, it determines our further strategy. First, we determine the energy spectra of the DHO, and then we calculate the proton transition rate through the IHO using the FGR.^22^ The Weyl symbol of the Hamiltonian for the DHO has the form

$$H_{W}^{\left(F,l\right)}(x,p)=\frac{p^{2}}{2m}+\frac{1}{2}m(\omega^{\left(F,l\right)}{)}^{2}(x-X^{\left(F,l\right)}{)}^{2}$$ 6

For simplicity, we use the following auxiliary notation: the terms ω^(*F*,*l*)^, *X* ^(*F*,*l*)^ are denoted by ω, *X*, respectively for a given form (*F*) and base pair (*l*). As this is a quadratic form in both positions, *x*, and momentum, *p*, hence the time-independent Moyal equation ([Disp-formula eq2]) takes the following form

$$\left[\frac{p^{2}}{2m}+\frac{1}{2}m\omega^{2}(x-X{)}^{2}-\frac{m\hbar \omega^{2}}{8}\partial_{p}^{2}-\frac{\hbar^{2}}{8m}\partial_{x}^{2}+i\left(m\hbar \omega^{2}(x-X)\partial_{p}-\frac{\hbar p}{2m}\partial_{x}\right)\right]\varrho (x,p)=(E-E^{\left(F,l\right)})\varrho (x,p)$$ 7

and its normalized solution has the form

$$\varrho_{n}(x-X,p)=\frac{(-1{)}^{n}}{\pi \hbar }\exp\left[-\left(\frac{m\omega }{\hbar }\right)(x-X{)}^{2}-(\hbar m\omega {)}^{-1}p^{2}\right]\times L_{n}\left(\frac{2m\omega (x-X{)}^{2}}{\hbar }+\frac{2p^{2}}{m\omega \hbar }\right)$$ 8

where *L*_*n*_(·) is the Laguerre polynomial of order *n*. In fact, the WDF given by eq 8 corresponds to the WDF for the fixed form *F* and the base pair *l*. For simplicity, we applied the following notation: ϱ_*n*_(·, ·) = ϱ_*n*_^(*F*,*l*)^(·, ·). Details of this solution are presented in the Supporting Information. In turn, the energy spectrum of the DHO has the form: *E*_*n*_^(*F*,*l*)^ = *ℏ*ω^(*F*,*l*)^(*n* + 1/2) + *E* ^(*F*,*l*)^, where *n* = 0, 1, ···. However, in our case, the quantum number *n* is finite for all considered cases, i.e., we assume that the individual DHOs are treated as independent finite-state systems. The present studies assume that the canonical and tautomeric forms contain *N* ^(C,A–T)^ = 7 and *N* ^(T,A–T)^ = 3 levels, respectively, for the A–T base. In contrast, we take *N* ^(C,G–C)^ = 9 and *N* ^(T,C–G)^ = 3 levels in the canonical and tautomeric forms for the G–C base, respectively. The potential energy profile for A–T and G–C base pairs within the harmonic approximation, together with energy spectra, is visualized in the Figure 2. In this Figure, we also marked the energies *E*_B_^*l*^ related to the height of the potential barriers, energy offsets, Δ*E* ^*l*^, that correspond to the minimum of the tautomeric wells. These are fixed relative to the minimum of the canonical wells for the base pairs under consideration. In turn, for them, minimum energies are set up as zero. Let us note that, despite the similarity of these both profiles, the height of the potential barrier of the A–T base pair, *E*_B_^A–T^, is higher than potential barrier of the G–C base pair, i.e., *E*_B_^A–T^ > *E*_B_^G–C^. Furthermore, the energy difference between the minima of the canonical and tautomeric forms for these two base pairs satisfy inequality (Δ*E* ^A–T^ > Δ*E* ^G–C^), and also the canonical and tautomeric forms in the A–T base pair are closer together (∼4 au) than in the G–C base pair (∼4.4 au).

**Figure 2:**
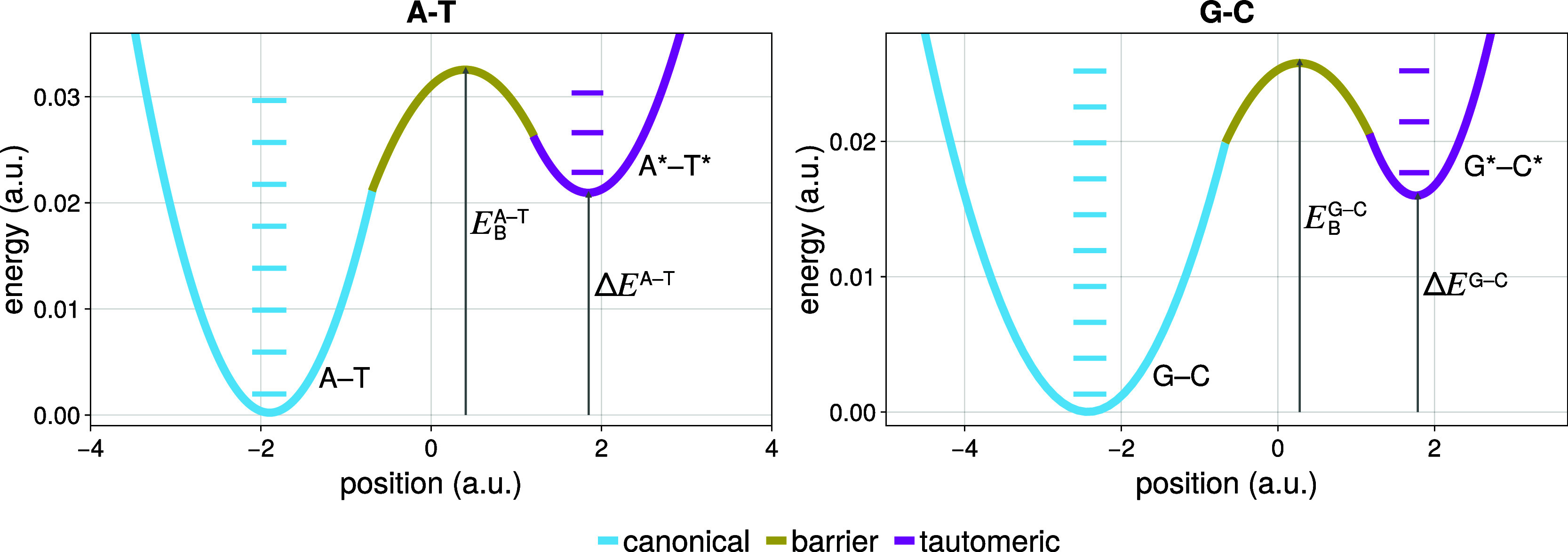
Potential energy profiles for the base pairs within the harmonic approximation: left panel: *l* = A–T; right panel: *l* = G–C. For each of the forms, there are depicted energy levels *E*_*n*_^(*F*,*l*)^. Symbols *E*_B_^*l*^ and Δ*E* ^*l*^ represent the height of the harmonic barrier and the energy difference between the forms, respectively.

At the end of these theoretical considerations, it is also worth exploring the generalization of the proposed model to the case of a two-proton transfer. In light of recent reports, this process is susceptible to environmental influences.^23,24^ For example, in G–C base pair, the presence of the helicase environment makes the tautomeric state less stable and thus reduces the probability of mutations. On the other hand, in the polymerase environment, the two-proton transfer associated with G–T tautomerization differs from that in aqueous and duplex environments, leading to G–T mispair tautomerization.

One of the most straightforward approaches to this problem involves including the effective potential, *V*_eff_(*x*), in the form of

$$V_{\mathrm{eff}}(x_{1})=\int_{R^{2}}dx_{2}dp_{2}U(x_{1},x_{2})\varrho^{\left(2\right)}(x_{1},p_{1};x_{2},p_{2})$$ 9

in the time-independent Moyal equation for a single proton. In this formula, *U*(*x*_1_, *x*_2_) denotes the potential energy of the two-proton interaction, and ϱ^(2)^(*x*_1_, *p*_1_;*x*_2_, *p*_2_) is the two-particle WDF. In the first approximation, the two-particle WDF can be replaced by the product of the one-particle WDF, and, as a result, the effective potential takes the form

$$V_{\mathrm{eff}}(x_{1})=\left[\int_{R^{2}}dx_{2}dp_{2}U(x_{1},x_{2})\varrho (x_{2},p_{2})\right]\varrho (x_{1},p_{1})$$ 10

which is equivalent to the mean field approximation. We proceed similarly with the second proton. Because each of the protons satisfies a single-particle time-independent Moyal equation with an effective potential generated self-consistently by the other proton’s WDF, this leads to the self-consistent procedure consisting of two single-particle Moyal equations. Let us note that the proposed generalization is based on a system of two integro-differential equations, which can only be solved using a numerical method.

## Results and Discussion

One of the primary approaches to determining the rate of the single-proton tunnelling process is based on the conventional transition–state theory,^25–27^ according to which, the rate coefficient of a first-order reaction is calculated by the formula^18^

$$k_{f}(T)=2\pi \kappa (T)\frac{k_{B}T}{\hbar }\exp\left[-\frac{\Delta E_{f}}{k_{B}T}\right]$$ 11

where Δ*E*_f_ is the activation energy for the reaction, *k*_B_ is the Boltzmann constant, *T* is the temperature, and κ­(*T*) is the temperature-dependent tunnelling coefficient. On the other hand, on the grounds of quantum theory, the transition rate of the proton between base pairs can be directly determined by the application of the FGR. Customizing this tool to the phase-space representation enables us to express the FGR formula for the discrete spectrum in the following form

$$\Gamma^{\left(l\right)}(\varepsilon )=\sum_{k=0}^{N^{\left(T,l\right)}}\sum_{j=0}^{N^{\left(C,l\right)}}\Gamma_{jk}^{\left(l\right)}(\varepsilon )$$ 12

where Γ_*jk*_^(*l*)^(ε) are the partial transition rates for the base pair *l* = A–T or G–C. The general form of this quantity is given by the formula^28^

$$\Gamma_{jk}^{\left(l\right)}(\varepsilon )=\frac{2\pi }{\hbar }\int_{R}dE2\pi \hbar \int_{R^{2}}dxdp\varrho_{j}^{\left(C,l\right)}(x,p)\varrho_{k}^{\left(T,l\right)}(x,p)|U^{\left(B,l\right)}(x){|}^{2}\times {\tilde{\delta }}_{\varepsilon }(E_{k}^{\left(T,l\right)}-E_{j}^{\left(C,l\right)}-E)$$ 13

where ϱ_*j*_^(C,*l*)^(*x*, *p*) and ϱ_*k*_^(T,*l*)^(*x*, *p*) represent the WDF of the initial (canonical form) and final (tautomeric form) state in the base pair *l*, respectively. In turn, *U* ^(B,*l*)^(*x*) represents the potential energy of the parabolic barrier, corresponding to the Weyl Symbol ([Disp-formula eq5]), and its parameters are given in Table S1 in the Supporting Information. Finally, the symbol δ̃_ε_(·) is reserved for the nascent Dirac’s delta, i.e., lim_ε→0_δ̃_ε_(*x*) = δ­(*x*). For the purpose of the present study, the nascent function is taken in the Gaussian form δ̃_ε_(*x*) = (2πε)^−1/2^exp­[−*x* ^2^/(2ε^2^)] with the dispersion, denoted by ε, that is conceptualized as the temperature broadening parameter. The form of this parameter is as follows ε = *k*_B_ *T*, where the used symbols have the usual meaning. Simultaneously, we neglect the direct influence of temperature on the potential energy profile, i.e., we neglect the vibrations of the base pairs in both considered cases. Admittedly, this assumption leads to further simplification of the considered harmonic approximation; however, it provides a quantitative formulation of the rigid double harmonic model of DNA, which is essentially a first approximation of the harmonic model. According to the proposed approach, we determine the influence of the temperature on the transition rates for the single-proton tunnelling process in A–T and G–C base pairs by taking the temperature broadening parameter, ε = ε­(*T*), into account in the FGR formula ([Disp-formula eq12]). The results of such calculations are presented in Figure 3. Let us note that both profiles of the transition rates exhibit an asymmetry with respect to their maxima, wherein the maximum corresponding to single-proton tunnelling in base pair G–C is shifted toward lower temperatures. As a result of the performed calculations, these maxima correspond to the temperatures: *T* = 147 K and *T* = 300 K for the single-proton tunnelling process in the base pairs G–C and A–T, respectively. Furthermore, let us note that the transition in A–T base pair is less effective than in the second pair under consideration. The reaction rate for the A–T base–pair is an order of magnitude lower than for G–C, confirming that the tautomeric form of A–T is less stable and less probable.^15,30^ The asymmetric forms of the transition rate profiles can be approximated by the Fano line curves^31^ in the biological relevant temperature regime of DNA bases with the asymmetry parameter *q* = 0.68 for the A–T, and *q* = 1.49 for the G–C. These numerical values of the asymmetry parameters exceed 1/2 in both cases and differ approximately 46% from each other. Hence, it can be concluded that in both cases, the proton transfer between base pairs is a thermally assisted process rather than a direct transfer between the canonical and tautomeric forms on both sides of the barrier formed by the IHO. However, from the inequality *q* ^G–C^ > *q* ^A–T^, it can be deduced that the direct transfer of the proton between the pointed-out states in the G–C pair base is stronger than in the A–T pair base due to the geometric parameters of the barrier (thinner, lower and more symmetrical); nevertheless, both of them are dominated by the thermally assisted process.

**Figure 3:**
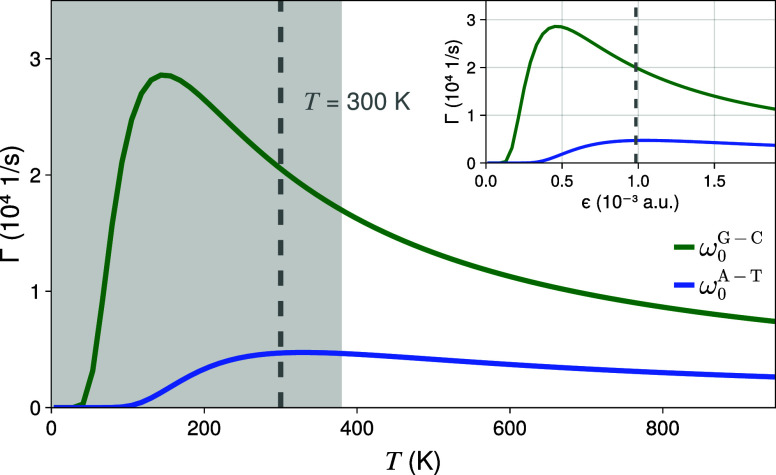
Transition rates derived from FGR for A–T and G–C base pairs as a function of temperature *T* or the temperature broadening ϵ. The biologically relevant temperatures are presented in color,^29^ and the temperature corresponding to 300 K is shown as a dashed line.

For the further study, we performed additional calculations of the proton transition rate based on the FGR within the considered model for different values of the angular frequency associated with the canonical wells, ω^(C,*l*)^, while the other two angular frequencies, ω^(T,*l*)^ and ω^(B,*l*)^, are fixed. This approach allows us to limit the number of model parameters. The results of calculations for the following set of angular frequencies {ω_0_, ω_1_, ω_2_, ω_3_, ω_4_} = {4.047, 4.112, 4.058, 4.003, 3.976} × 10^–3^ a.u. characterizing the canonical well in the A–T pair base, and the set {ω_0_, ω_1_, ω_2_, ω_3_, ω_4_} = {2.634, 2.696, 2.615, 2.593, 2.596} × 10^–3^ a.u. in the canonical well in the G–C pair base. Let us note that the angular frequency, ω_0_, is used as the reference frequency for both pairs, referring to previous results. The results are shown in Figure 4.

**Figure 4:**
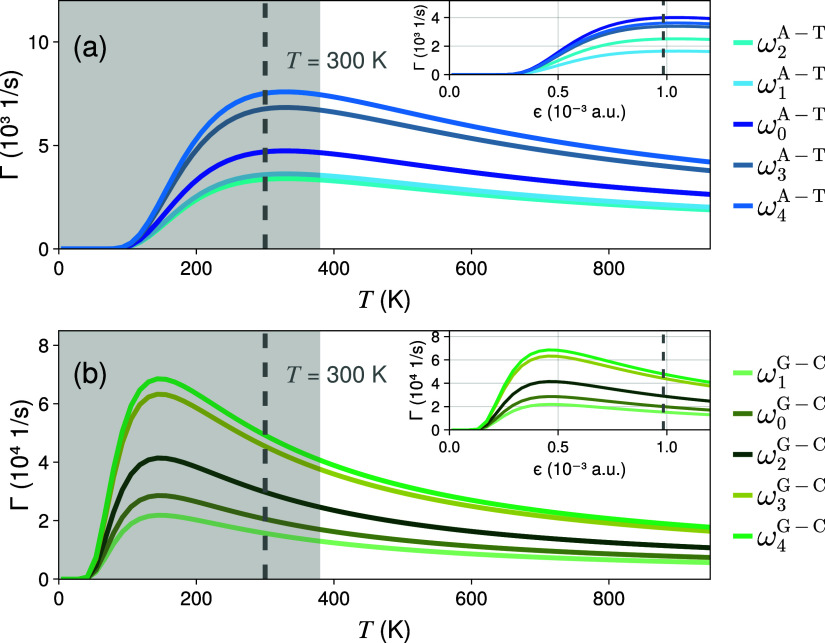
Transition rates derived from FGR for (a) A–T and (b) G–C base pair as a function of temperature or the temperature broadening parameter ϵ for different ω values. The biologically relevant temperatures are presented in color, and the temperature corresponding to 300 K is shown as a dashed line. The values ω_0_ = ω_0_^(*l*)^ for each base pair *l* are directly derived from the harmonic approximation from both potential energy formulas.^9,18^

According to these results, we observed that changes in the angular frequency of the canonical state within the considered range do not significantly alter the shape of the transition rate curves; they only increase their values. Thus, the asymmetry of the transition rate curves is conserved, and the Fano curve still reflects this behavior in both pair bases. To resume, it is essential to emphasize that, in the studies presented so far, the occupation probability of the energy levels in the DHO is identical for all initial states, and the temperature broadening associated with them supports the proton transition between the canonical and tautomeric forms. This consequently leads to the resonant and off-resonant contributions to the Fano line shape for the determined transition rates. This result is independent of the angular frequency characterizing the DHO of the canonical form.

In the following part of this study, we extend our analysis by assuming that the proton in the canonical wells is in thermal equilibrium with the environment at temperature *T*. This means that we now use the thermally averaged rate for the proton transition between the canonical and tautomeric forms. The thermally averaged FGR for the proton transition between the considered forms is calculated using the following formula

$${\tilde{\Gamma }}^{\left(l\right)}(\varepsilon )=\sum_{j=0}^{N^{\left(C,l\right)}}p_{j}(\varepsilon )\sum_{k=0}^{N^{\left(T,l\right)}}\Gamma_{jk}^{\left(l\right)}(\varepsilon )$$ 14

where *p*_*j*_(ε) is the Boltzmann weight assigned to the energy level *E*_*j*_ in the canonical wells, which has the form

$$p_{j}(\varepsilon )=\frac{\exp(-E_{j}/\varepsilon )}{\sum_{j=0}^{N^{\left(C,l\right)}}\exp(-E_{j}/\varepsilon )}$$ 15

This extension allows us to include the temperature effect on the single-proton transition between the considered base pairs and, simultaneously, to investigate the impact of the proposed extension of the FGR on the Fano-shaped temperature dependence. Using the formula ([Disp-formula eq14]), we calculate the thermal-averaged transition rates for different values of the angular frequencies associated with the canonical state of the considered base pairs, i.e., we use the same angular frequencies as in the previous case. The results are shown in Figure 5.

**Figure 5:**
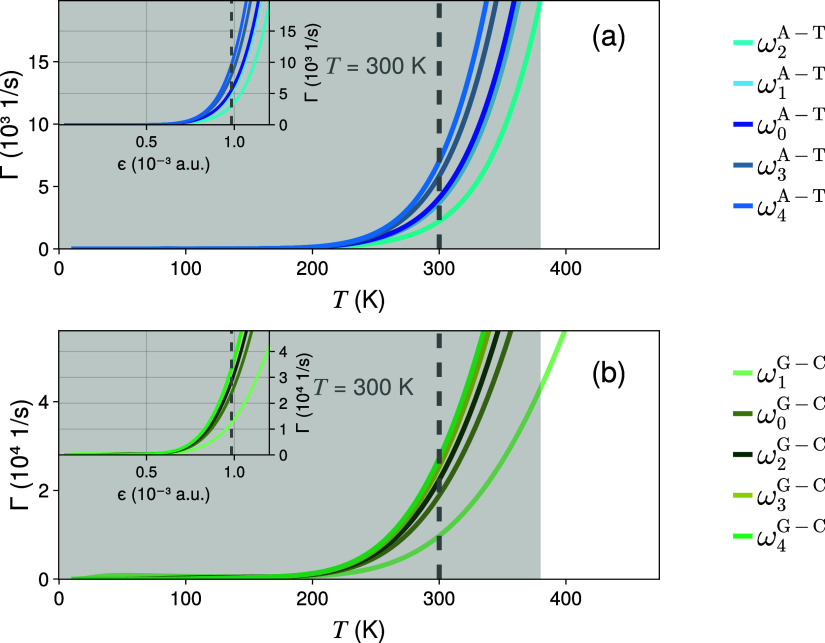
Transition rates derived from FGR with thermal coefficients for (a) A–T and (b) G–C base pair as a function of temperature or the temperature broadening parameter ϵ for different ω values. The biologically relevant temperatures are presented in color, and the temperature corresponding to 300 K is shown as a dashed line. The values ω_0_ = ω_0_^(*l*)^ for each base pair *l* are directly derived from the harmonic approximation from both potential energy formulas.^9,18^

Let us note that the curves obtained are no longer Fano-shaped; instead, they exhibit exponential growth with temperature (activation-like curves). This observation clearly indicates a strong dependence of the transition rate on thermally activated states, and particularly the higher states, whose contributions increase with increasing temperature. These high-energy level states lie closer to the barrier top, leading to much higher tunnelling probabilities. They dominate the total transition rate, leading to a monotonically increasing proton transition rate between the canonical and tautomeric forms in A–T and G–C base pairs over the biologically relevant temperature range.

For the quantitative comparison of the obtained transition rate results with those of other groups,^10,13,16,18^ we limited ourselves to presenting the results at temperature *T* = 300 K obtained within our model. In Table 1, we collected the numerical values of reaction and transition rates to compare them.

**Table 1:** Comparison of Reaction Rates *k*_f_ and Transition Rates Γ^(*l*)^ = Γ^(*l*)^(ω^(*l*)^) and Γ̃^(*l*)^ = Γ̃^(*l*)^(ω̃^(*l*)^) for Both Base Pairs^a^

*l*	*k*_f_ (s^–1^)	*k*_f_ (s^–1^)	*k*_f_ (s^–1^)	*k*_f_ (s^–1^)	Γ^(*l*)^ (s^–1^)	Γ̃^(*l*)^
A–T	7.17 × 10^3^	7.61 × 10^5^			7.55 × 10^3^	7.02 × 10^3^
G–C	2.83 × 10^4^	2.06 ± 3.38 × 10^6^	(5.30 ± 7.10) × 10^3^	≈ 10^4^	2.03 × 10^4^	2.64 × 10^4^
in	ref 16	ref 18	ref 13	ref 10		

a The frequencies used are ω^G–C^ = ω_4_^G–C^, ω̃^G–C^ = ω_0_^G–C^, ω^A–T^ = ω_3_^A–T^, ω̃^A–T^ = ω_3_^A–T^.

Let us note that the calculated transition rates, Γ^(*l*)^ and Γ̃^(*l*)^ at 300 K (last two columns in Table 1), for both DNA base pairs within the rigid harmonic model are consistent with Umesaki’s results,^16^ and Florian’s result^10^ for the G–C base pair. In turn, as we suppose, the discrepancy in the outcomes reported by Slocombe et al.^18^ (2 orders of magnitude) is due to (a) the inclusion of potential energy of the proton in the form of the nonharmonic profiles, and (b) the inclusion of dissipation and decoherence terms in the Moyal equation of motion for the WDF to model a thermal bath within the Caldeira–Leggett model, adapted to biologically relevant bath temperatures. In turn, our calculations are performed for a closed system; i.e., the temperature effect is accounted for solely by the thermal broadening of the states, with Boltzmann weights assigned to them to mimic the interaction of the DNA base with the thermal environment. However, this does not change the fact that, even in the simplest model of DNA, the environment’s influence cannot be ignored. Unless one considers a completely isolated strand of DNA at extremely low temperatures.

Finally, we determine the kinetic isotope effect (KIE), which provides a practical test of the presented double harmonic oscillator model of DNA for proton transfer. Let us define KIE in two ways using the ratios of the reaction rates for proton and deuteron for a given base pair *l*, separately, at temperature *T* = 300 K. In the first case, we use the bare FGR given by eq 12. According to this one, the KIE is expressed by the formula

$${\mathrm{KIE}}^{\left(l\right)}:=\frac{\Gamma^{\left(l\right)}(300\;K;\;\mathrm{proton})}{\Gamma^{\left(l\right)}(300\;K;\;\mathrm{deuteron})}$$ 16

In turn, for the second case, the definition of the KIE is obtained from the thermally averaged FGR formula ([Disp-formula eq14]), namely

$${\tilde{\mathrm{KIE}}}^{\left(l\right)}:=\frac{{\tilde{\Gamma }}^{\left(l\right)}(300\;K;\;\mathrm{proton})}{{\tilde{\Gamma }}^{\left(l\right)}(300\;K;\;\mathrm{deuteron})}$$ 17

For such defined KIEs, we obtained the following values within the formula ([Disp-formula eq16]): 48 and 45 for the A–T and G–C base pairs, respectively. In contrast, an application of formula ([Disp-formula eq17]) yields values of 28 and 31 for the A–T and G–C base pairs, respectively. The determined values of both kinetic isotope effects, i.e., KIE and $\tilde{\mathrm{KIE}}$ , for A–T and G–C base pairs at temperature *T* = 300 K are one order greater than can be expected in the case of the over-the-barrier mechanisms of transition due to purely thermal process. Such enhanced isotope sensitivity is consistent with proton transfer occurring via quantum tunnelling. The observed reduction of KIA from 48 to 28 for A–T base pair and from 45 to 31 for G–C base pair only indicates a strongly tunnelling-dominated mechanism at biologically relevant temperatures.^18^

## Concluding Remarks

In conclusion, we have developed an exactly solvable quantum-mechanical model of single–proton transfer between DNA base pairs within the phase-space representation of the quanta theory. This model is formed by the superposition of two rigid harmonic wells and one harmonic barrier which separates them. We have determined the transition rates of a proton between the canonical and tautomeric forms in the base pairs adenine – thymine and guanine – cytosine within this model by applying the Fermi Golden Rule. Within this approach, we have studied the single-proton transfer in two cases. In the first case, we have assumed that the temperature blurring of all energy levels is uniform to within *k*_B_ *T* and that these levels participate with equal weight in the calculation of the transition rate based on the Fermi Golden Rule. As a result, we have shown that the transition rates derived within this case (called ‘bare’ Fermi Golden Rule can be approximated by the Fano line curves in the biologically relevant temperature regime of the considered pair bases. In the second case, we have taken into account the thermal occupation of states using Boltzmann weights in the Gibbs canonical ensemble. Following this approach, we have observed that the Fano-type form of the transition rates transforms into exponential growth for the adenine-thymine and guanine-cytosine base pairs in the considered temperature regime. In this case, the exponential growth of the transition rates of the proton between the canonical and tautomeric forms emerges. This result provides strong evidence that the properties of the DNA depend on the environment represented by the thermal bath.

Additionally, we have determined the kinetic isotope effects for single-proton transfer between the canonical and tautomeric forms of the considered pair bases within the proposed model. The calculated values of these quantities for both bases, based on the Fermi Golden Rule, exceed the classical limit and therefore indicate that a genuinely tunnelling process governs the single-proton transfer in DNA base pairs. We have also shown that thermal averaging of the Fermi Golden Rule reduces the kinetic isotope effects for both pairs of bases. However, the main conclusion concerning the proton tunnelling process remains unchanged. Finally, we have estimated the kinetic isotope effect for the base pairs in question at room temperature using the ‘bare’ and thermally averaged Fermi Golden Rule, obtaining reasonable values in both cases. The numerical values of the calculated kinetic isotope effect, in both cases, allow one to conclude that the one-proton transition between the canonical and tautomeric forms in the base pairs adenine-thymine and guanine-cytosine is dominated by tunnelling at biologically relevant temperatures.

## Supplementary Material



## Notes

Detailed derivations of some theoretical results and additional results related to the numerical methods applied in this study (PDF)

## References

[1] Watson J. D., Crick F. H. C. (1953). The structure of DNA. Cold Spring
Harb. Symp. Quant. Biol..

[2] Cobb M., Comfort N. (2023). What Rosalind Franklin truly contributed
to the discovery
of DNA’s structure. Nature.

[3] Löwdin P. O. (1963). Proton
Tunneling in DNA and its Biological Implications. Rev. Mod. Phys..

[4] Gheorghiu, A. Ensemble-based multiscale modelling of DNA base pair tautomerism in the absence and presence of external electric fields, Ph.D. Thesis; Department of Chemistry University College London: London, UK, 2021.

[5] Schrödinger, E. What is Life? With Mind and Matter and Autobiographical Sketches; Cambridge University Press: Cambridge, 2012.

[6] Brookes J.
C. (2017). Quantum
effects in biology: golden rule in enzymes, olfaction, photosynthesis
and magnetodetection. Proc. R. Soc. A.

[7] McFadden J., Al-Khalili J. (2018). The origins of quantum biology. Proc. R. Soc. A.

[8] Kim Y., Bertagna F., D’Souza E. M., Heyes D. J., Johannissen L. O., Nery E. T., Pantelias A., Sanchez-Pedreño J. A., Slocombe L., Spencer M. G., Al-Khalili J., Engel G. S., Hay S., Hingley-Wilson S. M., Jeevaratnam K., Jones A. R., Kattnig D. R., Lewis R., Sacchi M., Scrutton N. S., Silva S., Ravi P., McFadden J. (2021). Quantum Biology: An Update and Perspective. Quantum Rep..

[9] Godbeer A. D., Al-Khalili J. S., Stevenson P. D. (2015). Modelling proton tunnelling in the
adenine-thymine base pair. Phys. Chem. Chem.
Phys..

[10] Florián J., Leszczyński J. (1996). Spontaneous
DNA Mutations Induced by Proton Transfer
in the Guanine·Cytosine Base Pairs: An Energetic Perspective. J. Am. Chem. Soc..

[11] Slocombe L., Al-Khalili J. S., Sacchi M. (2021). Quantum and classical effects in
DNA point mutations: Watson–Crick tautomerism in AT and GC
base pairs. Phys. Chem. Chem. Phys..

[12] Slocombe L., Winokan M., Al-Khalili J., Sacchi M. (2022). Proton transfer during
DNA strand separation as a source of mutagenic guanine-cytosine tautomers. Commun. Chem..

[13] Gheorghiu A., Coveney P. V., Arabi A. A. (2021). The influence
of external electric
fields on proton transfer tautomerism in the guanine–cytosine
base pair. Phys. Chem. Chem. Phys..

[14] Gheorghiu A., Coveney P. V., Arabi A. A. (2020). The influence
of base pair tautomerism
on single point mutations in aqueous DNA. Interface
Focus.

[15] Arabi A. A., Matta C. F. (2018). Adenine–thymine
tautomerization under the influence
of strong homogeneous electric fields. Phys.
Chem. Chem. Phys..

[16] Umesaki K., Odai K. (2020). A Kinetic Approach to Double Proton Transfer in Watson–Crick
DNA Base Pairs. J. Phys. Chem. B.

[17] Slocombe L., Winokan M., Al-Khalili J., Sacchi M. (2023). Quantum Tunnelling
Effects in the Guanine-Thymine Wobble Misincorporation via Tautomerism. J. Phys. Chem. Lett..

[18] Slocombe L., Sacchi M., Al-Khalili J. (2022). An open quantum
systems approach
to proton tunnelling in DNA. Commun. Phys..

[19] Warman H., Slocombe L., Sacchi M. (2023). How proton
transfer impacts hachimoji
DNA. RSC Adv..

[20] Tosiek J. (2012). The eigenvalue
equation for a 1-D Hamilton function in deformation quantization. Phys. Lett. A.

[21] Hug M., Menke C., Schleich W. P. (1998). Modified
spectral method in phase
space: Calculation of the Wigner function. I.
Fundamentals. Phys. Rev. A.

[22] Białynicki-Birula, I. ; Cieplak, M. ; Kamiński, J. Theory of Quanta; Oxford University Press, Inc.: 1992.

[23] Winokan M., Slocombe L., Al-Khalili J., Sacchi M. (2023). Multiscale simulations
reveal the role of PcrA helicase in protecting against spontaneous
point mutations in DNA. Sci. Rep..

[24] Li P., Rangadurai A., Al-Hashimi H. M., Hammes-Schiffer S. (2020). Environmental
Effects on Guanine-Thymine Mispair Tautomerization Explored with Quantum
Mechanical/Molecular Mechanical Free Energy Simulations. J. Am. Chem. Soc..

[25] Eyring H. (1935). The Activated
Complex in Chemical Reactions. J. Chem. Phys..

[26] Laidler K., King M. C. (1983). Development of transition-state
theory. Jo. of Phys. Chem..

[27] Truhlar D. G., Hase W. L., Hynes J. T. (1983). Current
status of transition-state
theory. J. Phys. Chem..

[28] The derivation of the FGR in the phase-space representation is presented in Section S3 of the Supporting Information.

[29] Marguet E., Forterre P. (1994). DNA stability at temperatures
typical for hyperthermophiles. Nucleic Acids
Res..

[30] Fu L.-Y., Wang G.-Z., Ma B.-G., Zhang H.-Y. (2011). Exploring the common
molecular basis for the universal DNA mutation bias: Revival of Löwdin
mutation model. Biochem. Biophys. Res. Commun..

[31] Rau A. R. P. (2004). Perspectives
on the Fano Resonance Formula. Phys. Scr..

