# Supplementary material for: The Rigid Double Harmonic Oscillator Model of DNA Base Pairs and the Wignerian Approach for the Transition Rate of Single-Proton Transfer within the Fermi Golden Rule
Source: J Phys Chem B. 2026 Feb 19;130(9):2531–8. doi: 10.1021/acs.jpcb.5c08297 (PMC13298915; doi:10.1021/acs.jpcb.5c08297)
Supplement: Supplementary file 1 [file jp5c08297_si_001.pdf]

Supporting Information for The Rigid Double Harmonic Oscillator Model of DNA Base Pairs and the Wignerian Approach for the Transition Rate for Single-Proton Transfer within the Fermi Golden Rule

M. Wleklińska[1] and B. J. Spisak*[1]

[1]AGH University of Krakow, Faculty of Physics and Applied Computer Science, al. A. Mickiewicza 30, 30-059 Krakow, Poland

This Supporting Information for the research entitled 'The rigid double harmonic oscillator model of DNA base pairs and the Wignerian approach for the transition rate for single-proton transfer within the Fermi Golden Rule' contains additional results from our studies in the form of detailed derivations and data directly related to the numerical determination of the potential and Fano profile.

S1 Potential Energy Profile of the DNA Nitrogenous Bases within the Harmonic Approximation

The potential energy profiles of the DNA bases for the pairs A–T and G–C were determined by using DFT calculations performed by Godbeer et al. for the A–T base pair [1] and by Slocombe et al. for the G–C base pair [2]. These results are consistent with the fourth-order polynomial in the former case and the double Morse function in the latter case. The primary difficulty with these types of functions representing energy potentials is that their use in constructing exactly solvable Hamiltonians, in the sense of precisely determining their spectral properties—such as the energy spectrum and the corresponding eigenfunctions—presents a challenge at the current stage of knowledge. Hence, we proposed simple, but not overly simplistic, models of the potential energy profiles based on the harmonic approximation of DFT results. To ensure their optimal compatibility, we numerically approximated the potential energy profiles derived from DFT calculations conducted by the authors mentioned above. The approximation was carried out in `Python` and `Julia` code that utilised numerical packages implementing the least squares method.

*email: bjs@agh.edu.pl

The source codes are available from the corresponding author upon reasonable request. Fig. S1 shows the comparison of the DFT results with the results obtained within the least squares method. The approximate energy profile potential (three-colour solid line)

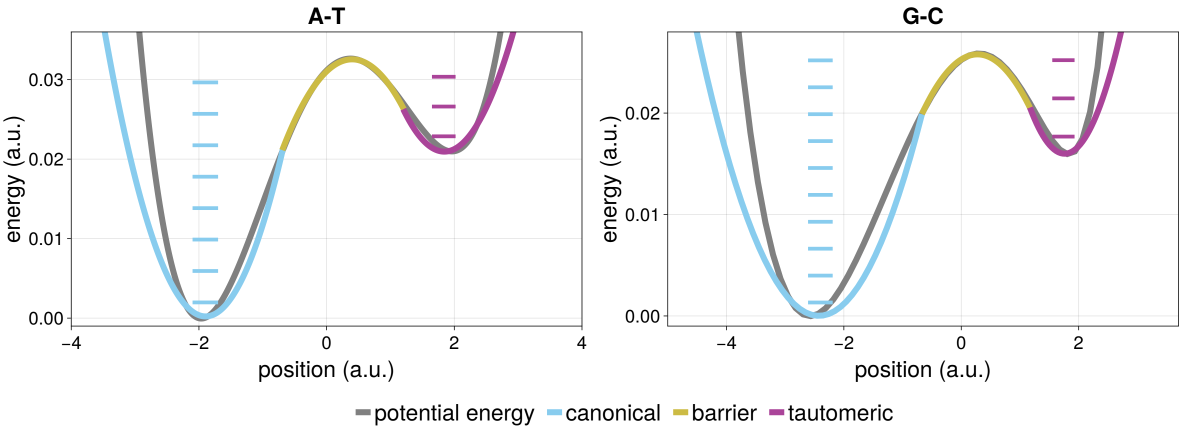

Figure S1: Potential energy harmonic approximation with the original potential energy graphs derived from left: [1] for the A–T base pair and right: [3] for the G–C base pair.

presented in this figure is described by a 2nd-order polynomial, in the following form

$$U(x) = \sum_{r=0}^{2} a_r^{(F,\,l)} x^r, \tag{S1}$$

where $F = \mathrm{C}, \mathrm{T}, \mathrm{B}$ denotes the state (C - canonical, T - tautomeric and B - barrier) and l corresponds to specific base pair, that is A–T or G–C. All parameters required for reproducing the double-well potential energy, determined through numerical approximation (the least squares method), are collected in the Tab. S1. In the figure S1, we also

Table S1: Quantitative parameter values of the second–order polynomials that describe the canonical (C), tautomeric (T) states and the barrier (B) used in the model for each base pair: A–T and G–C.

	$a_2^{(F,\,l)}$	$a_1^{(F,\,l)}$	$a_0^{(F,\,l)}$	x range
l = A–T				
**canonical form** (C)	0.0143	0.0545	0.0534	$(-\infty, -0.71)$
**barrier** (B)	-0.0096	0.0076	0.0310	$(-0.71, 1.17)$
**tautomeric form** (T)	0.0128	0.0128	0.0621	$(1.17, \infty)$
l = G–C				
**canonical form** (C)	0.0065	0.0313	0.0398	$(-\infty, -0.67)$
**barrier** (B)	-0.0065	0.0039	0.0250	$(-0.67, 1.15)$
**tautomeric form** (T)	0.0130	-0.0446	0.0547	$(1.15, \infty)$

depict the energy levels corresponding to each of the harmonic wells separately, i.e. the

energy spectra of the two wells are considered independent [cf. Sec. S2]. In turn, the part of the potential energy represented by the parabolic barrier is treated as an obstacle that separates the harmonic wells. In the presented calculations, it is taken into account perturbatively by the application of the Fermi Golden Rule [cf. Sec. S3]. For the fixed pair of indices $(F,\ l)$, the formula given by eq (S1) can be written down in a more suitable form for our goal, namely

$$U^{(F,\,l)}(x) = a_2^{(F,\,l)} \left[x + \frac{a_1^{(F,\,l)}}{2a_2^{(F,\,l)}} \right]^2 + a_0^{(F,\,l)} - \frac{\left(a_1^{(F,\,l)}\right)^2}{4a_2^{(F,\,l)}}. \tag{S2}$$

After implementing the following auxiliary symbols: $X^{(F,\,l)} := a_1^{(F,\,l)}/(2a_2^{(F,\,l)})$ and $C^{(F,\,l)} := a_0^{(F,\,l)} - (a_1^{(F,\,l)})^2/(4a_2^{(F,l)})$, the considered formula takes the form

$$U^{(F,\,l)}(x) = a_2^{(F,\,l)} \left[x + X^{(F,\,l)}\right]^2 + C^{(F,\,l)}, \tag{S3}$$

where $C^{(F,\,l)}$ represents an energy offset , which can be specified with the symbol ΔE^l, and $X^{(F,\,l)}$ is the location of the local extrema for the parabolic potential, i.e. local minima for wells and local maximum for the harmonic barrier. In turn the coefficient $a_2^{(F,\,l)}$ can be identified as the $m[\omega^{(F,\,l)}]^2/2$. Since a polynomial of the second order represents each of these three potential energy terms separately, and collectively they constitute a continuous form of the double potential energy profile for a proton as a function of the position variable. In consequence, the total potential energy profile consists of the two displacement harmonic oscillators separated by the inverse harmonic oscillator. Therefore, this approach is called the rigid double harmonic oscillator model. Based on this consideration of the potential energy profile presented above, we designed the rigid double harmonic oscillator of the DNA nitrogen bases as follows. Within this approach, we start by defining the Weyl symbol of the total Hamiltonian, $\widetilde{H}_W^{(\widetilde{F},l)}(x,p)$ for the system in question, with fixed pair $(\widetilde{F}, l)$, which is expressed by the formula

$$\widetilde{H}_{\rm W}^{(\widetilde{F},l)}(x,p) = \frac{p^2}{2m} + \sum_{r=0}^{2} a_r^{(\widetilde{F},\,l)} x^r \tag{S4}$$

where the coefficients $a_r^{(\widetilde{F},\,l)}$ are collected in Tab. S1, and $\widetilde{F} = \mathrm{C}, \mathrm{T}$, and B. In the proposed approximation, we replace the Weyl symbol of the total Hamiltonian, $\widetilde{H}_{\rm W}^{(\widetilde{F},\,l)}(x,p)$ with two separate Weyl symbols, one corresponding to the Hamiltonian, $H_{\rm W}^{(\mathrm{C},l)}(x,p)$, of the canonical form and the other to the Hamiltonian, $H_{\rm W}^{(\mathrm{T},\,l)}(x,p)$ of the tautomeric form. In turn, the part of the original potential energy that combines these two forms is extracted as a potential barrier, thereby constituting the perturbation. According to the proposed separation, the Weyl symbol of the Hamiltonian, $H_{\rm W}^{(F)}(x,p)$, for the canonical and tautomeric forms ($F = \mathrm{C}$ and T, respectively), with a fixed l, is given by the formula

$$H_{\rm W}^{(F,l)}(x,p) = \frac{p^2}{2m} + \frac{1}{2} m [\omega^{(F,\,l)}]^2 \left[x + X^{(F,\,l)}\right]^2 + C^{(F,\,l)}, \tag{S5}$$

whereas the Weyl symbol of the perturbation representing the potential barrier, $U^{(\mathrm{B},\,l)}(x)$, in both base pairs $l = \mathrm{A} - \mathrm{T},\ \mathrm{G} - \mathrm{C}$ is taken into account separately. The main idea of the presented approach is illustrated in Fig. S2 in the form of the energy surface of

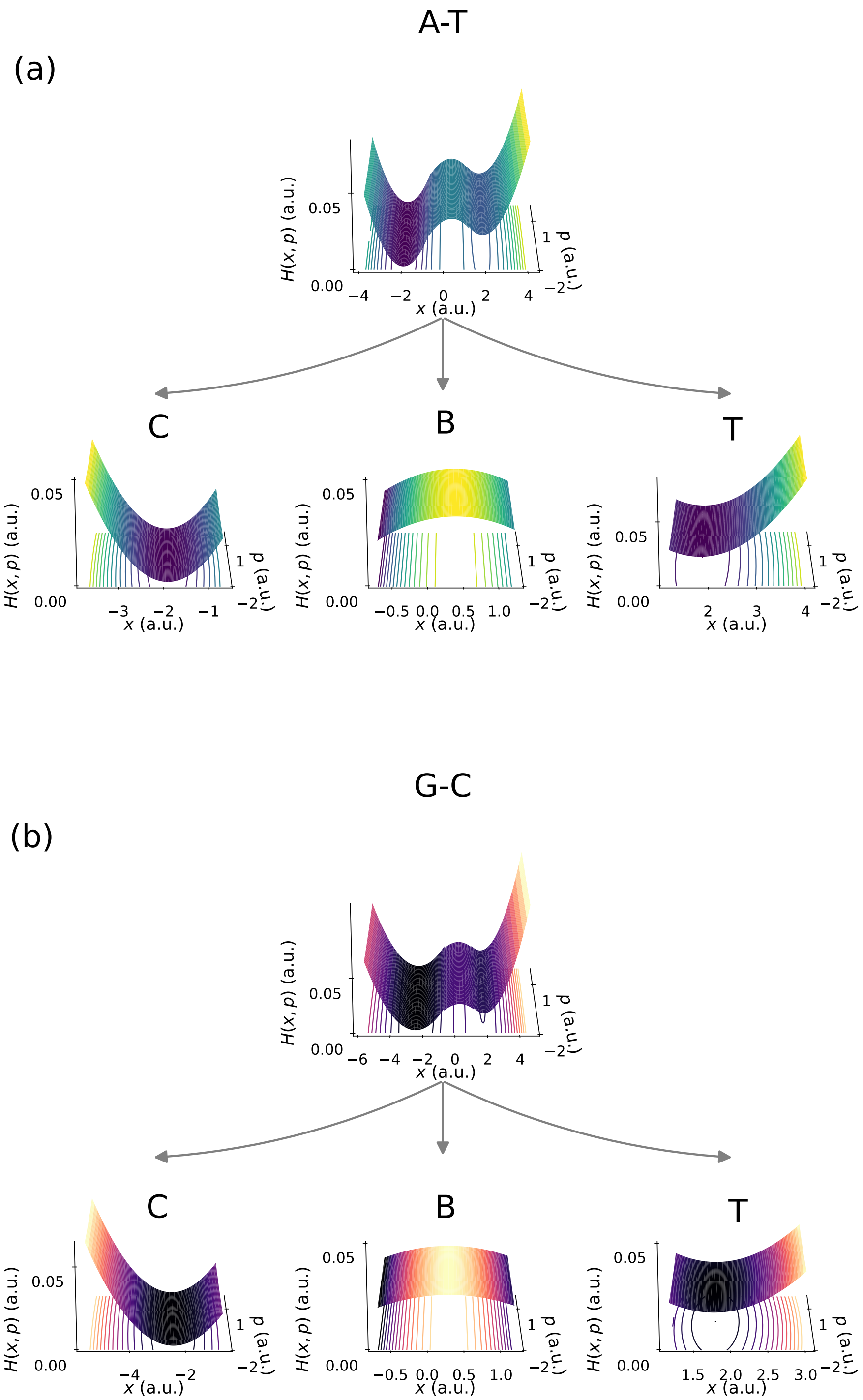

Figure S2: Illustration of the division of the input Weyl symbol of the Hamiltonian into three partial Weyl symbols associated with the canonical (C), tautomeric (T) forms, and the barrier (B) region. These forms constitute independent elements that are utilised to define the model of the DNA base pairs: (a) A–T and (b) G–C. The Weyl symbols are represented by the energy surfaces, which are real polynomials of the second order over the phase space spanned by the position and momentum variables. The contour lines represent the equipotential lines of the Weyl symbols of the systems under consideration.

the Weyl symbol of the Hamiltonians $\widetilde{H}_W^{(\widetilde{F},l)}(x,p)$ and $H = H_W^{F,l}(x,p)$, for the DNA base pairs (a) A − T, (b) G − C, separately. As illustrated by the diagrams, the input Weyl symbol of the Hamiltonian for the double harmonic-well system can be decomposed into three distinct terms. These terms consist of two independent displacement harmonic oscillators and an inverted harmonic oscillator.

S2 Solution of the Star-Genvalue Equation for the Displacement Harmonic Oscillator

Let us note that the determination of the WDF within the Wignerian approach is based on the solution of the following $\star$-genvalue equation

$$\left[\left(\frac{p^2}{2m} + U_{\mathrm{W}}\right) \star \varrho\right](x,p) = E\varrho(x,p). \tag{S6}$$

In fact, this equation, for a potential energy, $U(x)$, belonging to the class of n-differentiable real functions, $U(x) \in C^n(\mathbb{R})$, can be converted into the partial differential equation of the n-th order. It stems from the fact that the exponential operator [cf. eq (4)] is represented by the following series [4]

$$\exp\left[\frac{\mathrm{i}\hbar}{2}\left(\overleftarrow{\partial_x}\overrightarrow{\partial_p} - \overleftarrow{\partial_p}\overrightarrow{\partial_x}\right)\right] = \lim_{n\to\infty}\sum_{k=0}^{n}\frac{(\mathrm{i}\hbar)^k}{2^k k!}\left(\overleftarrow{\partial_x}\overrightarrow{\partial_p} - \overleftarrow{\partial_p}\overrightarrow{\partial_x}\right)^k. \tag{S7}$$

Substituting series given by eq (S7) into eq (S6), we obtain, after some tedious but not-so-difficult manipulations, the following partial differential equation

$$\frac{1}{2m}\left(p^2 - \mathrm{i}\hbar p - \frac{\hbar^2}{4}\partial^2_{xx}\right)\varrho(x,p) + \lim_{n\to\infty}\sum_{k=0}^{n}\frac{(\mathrm{i}\hbar)^k}{2^k k!}U_{\mathrm{W}}(x)\left(\overleftarrow{\partial_x}\overrightarrow{\partial_p} - \overleftarrow{\partial_p}\overrightarrow{\partial_x}\right)^k\varrho(x,p). \tag{S8}$$

The derived equation can be significantly simplified for potential energies represented by low-order polynomial functions. This is particularly evident for quadratic functions, which correspond to our problem rigorously because the $\star$-genvalue equation reduces itself to the partial differential equation of the second order, which can be solved exactly.

Structurally, the Weyl symbols for the Hamiltonians $H_{\mathrm{W}}^{(\mathrm{C})}$ and $H_{\mathrm{W}}^{(\mathrm{T})}$ corresponding to the potential wells are similar, and their form follows

$$H_{\mathrm{W}}^{(F,\,l)}(x,p) = \frac{p^2}{2m} + \frac{1}{2}m\left(\omega^{(F,\,l)}\right)^2\left(x - X^{(F,\,l)}\right)^2. \tag{S9}$$

For simplicity, we use the following auxiliary notation: the terms $[\omega^{(\mathrm{F},\,l)}]$, $X^{(F,\,l)}$ are denoted by ω, X, respectively for a given form (F) and base pair (l). Substituting the potential part of the Weyl symbol given by eq (S9), i.e. the potential energy of the displacement harmonic oscillator, into the general form of the $\star$-genvalue equation (S8), we obtain the partial differential equation of the second-order, which is equivalent to the following system of two partial differential equations

$$\begin{cases} \left[p^2/(2m) + (m\omega^2/2)(x-X)^2 - (m(\hbar\omega)^2/8)\partial^2_{pp} - \hbar^2/(8m)\partial^2_{xx}\right]\varrho(x,p) = (E - E^{(F,\,l)})\varrho(x,p) \\ \left[m\hbar\omega^2(x-X)\partial_p - (\hbar p)/(2m)\partial_x\right]\varrho(x,p) = 0. \end{cases} \tag{S10}$$

As was mentioned in main body of the text, these two equations correspond to real and imaginary parts of the initial equation, which results from eq (S8) for the potential energy in the form of a quadratic function. We find the solution for this system in two steps. Firstly, by applying the method of characteristics [5], we determine the solution of the 1st-order partial differential equation

$$\left[m\hbar\omega^2(x-X)\partial_p - \frac{\hbar p}{2m}\partial_x\right] \varrho(x,p) = 0. \tag{S11}$$

This solution has the form

$$\varrho(x,p) = f(z) \tag{S12}$$

where $f(z)$ is an arbitrary function of the one scalar variable z that is given by the formula

$$z = \frac{4}{\hbar\omega}H(x,p) = \frac{4}{\hbar\omega}\left(\frac{p^2}{2m} + \frac{1}{2}m\omega^2(x-X)^2\right). \tag{S13}$$

On the other hand, the function $f(\cdot)$ must also satisfy the second-order partial differential equation in the form

$$\left[\frac{p^2}{2m} + \frac{1}{2}m\omega^2(x-X)^2 - \frac{m(\hbar\omega)^2}{8}\partial^2_{pp} - \frac{\hbar^2}{8m}\partial^2_{xx}\right] \varrho(x,p) = (E - E^{(F,\,l)})\varrho(x,p). \tag{S14}$$

We are seeking the solution of this equation within the class of twice differentiable functions. Substituting the variables (x,p) in eq (S14) with the variable z, in accordance with the formula (S13), we obtain the second-order partial differential equation in the form

$$\left(z - 4\partial_z - 4z\partial^2_{zz}\right) f(z) = \frac{4(E-C)}{\hbar\omega} f(z), \tag{S15}$$

where $C = C^{(F,\,l)}$ for a given form F and base pair l. This last equation can be solved by the Sommerfeld polynomial method, postulating the solution in the product form [6]

$$f(z) = \phi(z)e^{-z/2}, \tag{S16}$$

where the function $\phi(z)$ is a polynomial. Inserting the function $f(z)$ given by eq (S16) into differential equation (S15), we obtain the eigenvalue equation for the polynomial $\phi(z)$ in the form

$$z\partial^2_{zz}\phi(z) + (1-z)\partial_z\phi(z) + \varepsilon\phi(z) = 0, \tag{S17}$$

where ε is given by the formula

$$\varepsilon = \frac{E-C}{\hbar\omega} - \frac{1}{2}. \tag{S18}$$

For $\varepsilon = n$, where $n \in \{0, 1, \ldots, \}$, eq (S17) corresponds to the Laguerre equation [6] and its solution is given by the eigenfunction in the form of the n-th order Laguerre polynomial, i.e. $\phi(z) = L_n(z)$. Hence, we conclude that the solution (S16) takes the form

$$f(z) = L_n(z)e^{-z/2} \tag{S19}$$

and the corresponding eigenvalue, E_n is given by the formula

$$E_n = \hbar\omega(n + 1/2) + C. \tag{S20}$$

The symbol for the eigenenergy E_n corresponds to $E_n^{(F,\,l)}$ for fixed a form $F = \mathrm{C},\ \mathrm{T}$ and a given base pair $l = \mathrm{A-T},\ \mathrm{G-C}$ in the main body of the text.

S3 The Phase-Space Representation of the Fermi Golden Rule

The Fermi Golden Rule (FGR) was developed based on first-order time-dependent perturbation theory to determine the transition rates between quantum states under the influence of a perturbation. In this section, we will discuss the FGR representation in phase space, based on its textbook form [7]. Let us consider an isolated system for a fixed base pair l. The initial configuration relating to the canonical form is expressed by the state $|\phi_j^{(\mathrm{C},\,l)}\rangle$, to which corresponds the energy $E_j^{(\mathrm{C},\,l)}$, where j is finite. In turn, the state $|\psi_k^{(\mathrm{T},\,l)}\rangle$ represents the final configuration corresponding to the tautomeric form with the energy $E_k^{(\mathrm{T},\,l)}$, where k is finite. It is also relevant to mention that, in the explored model, we take into account that $j > k$. The transition probability per unit time interval due to the time-independent perturbation, $\hat{U}^{(\mathrm{B},\,l)}$, can be written in the symbolic form as follows [7]

$$\Gamma_{jk}^{(l)} = \frac{2\pi}{\hbar} \left| \left\langle \psi_k^{(\mathrm{T},\,l)} \middle| \hat{U}^{(\mathrm{B},\,l)} \middle| \phi_j^{(\mathrm{C},\,l)} \right\rangle \right|^2 \delta\left(E_k^{(\mathrm{T},\,l)} - E_j^{(\mathrm{C},\,l)} \right), \tag{S21}$$

where $\delta(\cdot)$ is the Dirac delta. Let us introduce the auxiliary symbol $\kappa_{kl}^{(l)}$ for denoting the matrix element of the perturbation according to the formula

$$\kappa_{kl}^{(l)} := \left\langle \psi_k^{(\mathrm{T},\,l)} \middle| \hat{U}^{(\mathrm{B},\,l)} \middle| \phi_j^{(\mathrm{C},\,l)} \right\rangle. \tag{S22}$$

Based on this notation, the considered transition probability, $\Gamma_{jk}^{(l)}$, takes the following form

$$\Gamma_{jk}^{(l)} = \frac{2\pi}{\hbar} \left| \kappa_{kl}^{(l)} \right|^2 \delta\left(E_k^{(\mathrm{T},\,l)} - E_j^{(\mathrm{C},\,l)} \right), \tag{S23}$$

By decomposing the square of the modulus of the matrix element, $\kappa_{kl}^{(l)}$, according to its definition and employing the conjugate symmetry property of the Hermitian scalar product, we can convert this quantity into the following form

$$\left| \kappa_{kl}^{(l)} \right|^2 = \langle \psi_k^{(\mathrm{T},\,l)} | \hat{U}^{(\mathrm{B},\,l)} | \phi_j^{(\mathrm{C},\,l)} \rangle \langle \phi_j^{(\mathrm{C},\,l)} | U^{(\hat{\mathrm{B}},\,l)} | \psi_k^{(\mathrm{T},\,l)} \rangle, \tag{S24}$$

and then, expressing the pure states through the density operators, $\hat{\rho}_r^{(F,\,l)}$, for $r = j, k$ and $F = \mathrm{C},\ \mathrm{T}$, we obtain

$$\left| \kappa_{kl}^{(l)} \right|^2 = \langle \psi_k^{(\mathrm{T},\,l)} | \hat{U}^{(\mathrm{B},\,l)} | \phi_j^{(\mathrm{C},\,l)} \rangle \langle \phi_j^{(\mathrm{C},\,l)} | \hat{U}^{(\mathrm{B},\,l)} | \psi_k^{(\mathrm{T},\,l)} \rangle = \mathrm{Tr}\left[\hat{\rho}_k^{(\mathrm{T},\,l)} \hat{U}^{(\mathrm{B},\,l)} \hat{\rho}_j^{(\mathrm{C},\,l)} \hat{U}^{(\mathrm{B},\,l)\dagger} \right], \tag{S25}$$

where the symbol $\dagger$ represents the Hermitian conjugation. For the operator $\hat{U}^{(\mathrm{B},\,l)}$ that is represented by a scalar-valued function $U(x)$, in the position representation, the expression given by equation (S25) can be reduced to the form

$$\left| \kappa_{kl}^{(l)} \right|^2 = \mathrm{Tr}\left[\hat{\rho}_k^{(\mathrm{T},\,l)} \left| \hat{U}^{(\mathrm{B},\,l)} \right|^2 \hat{\rho}_j^{(\mathrm{C},\,l)} \right]. \tag{S26}$$

Taking into account the perturbation energy operator expressed by a quadratic function in position representation, after the application of the Weyl transform, this expression takes the form

$$\mathrm{Tr}\left[\hat{\rho}_k^{(\mathrm{T},\,l)} \left| U^{(\mathrm{B},\,l)}(x) \right|^2 \hat{\rho}_j^{(\mathrm{C},\,l)} \right] = 2\pi\hbar \int_{\mathbb{R}^2} \mathrm{d}x\, \mathrm{d}p\ \left[U^{(\mathrm{B},\,l)}(x) \right]^2 \varrho_k^{(\mathrm{T},\,l)}(x,p) \varrho_j^{(\mathrm{C},\,l)}(x,p), \tag{S27}$$

where the product of the Wigner distribution functions associated with the initial and final states is used instead of the cross-Wigner distribution function. Let us observe that this type of approximation renders our approach semiclassical. Substituting the result given by equation (S27) into the expression given by equation (S26), and then incorporating it in the equation (S21), leads to the following formula for transition probability per unit time interval in the phase-space representation

$$\Gamma_{jk}^{(l)} = \frac{2\pi}{\hbar} 2\pi\hbar \int_{\mathbb{R}^2} \mathrm{d}x\mathrm{d}p \ \left[U^{(\mathrm{B},\, l)}(x)\right]^2 \varrho_k^{(\mathrm{T},\, l)}(x,p)\, \varrho_j^{(\mathrm{C},\, l)}(x,p) \delta\left(E_k^{(\mathrm{T},\, l)} - E_j^{(\mathrm{C},\, l)}\right) \tag{S28}$$

or, by extending this expression to include all possible energy transfers within the system, we perform an integration over energy, and consequently, the following formula emerges

$$\Gamma_{jk}^{(l)} = \frac{2\pi}{\hbar} \int_{\mathbb{R}} \mathrm{d}E \ 2\pi\hbar \int_{\mathbb{R}^2} \mathrm{d}x \ \mathrm{d}p \ \left[U^{(\mathrm{B},\, l)}(x)\right]^2 \varrho_k^{(\mathrm{T},\, l)}(x,p)\, \varrho_j^{(\mathrm{C},\, l)}(x,p) \delta\left(E_k^{(\mathrm{T},\, l)} - E_j^{(\mathrm{C},\, l)} - E\right). \tag{S29}$$

Hence, the total transition probability per unit time interval, $\Gamma^{(l)}$, for fixed base pairs l, which includes all possible initial and final quantum states that participate in transitions induced by the perturbation, has the form

$$\Gamma^{(l)} = \sum_j \sum_k \Gamma_{jk}^{(l)}. \tag{S30}$$

where $\Gamma_{jk}^{(l)}$ is given by equation (S29).

S4 Fano Line Curves of the Transition Rate Profiles

The transition rates as a function of the dimensionless energy parameter, ϵ, display asymmetry profiles for each of the considered base-pairs l = A–T. These profiles can be approximated by the Fano formula according to the following expression [8]

$$\Gamma^{(l)}(\epsilon) = \alpha^{(l)} \frac{\left(\epsilon + q^{(l)}\right)^2}{\epsilon^2 + 1}, \tag{S31}$$

where $\alpha^{(l)}$ is a scaling parameter, $q^{(l)}$ is the asymmetry parameter, and $\epsilon = (\varepsilon - \epsilon_0^{(l)})/(\gamma^{(l)})$ is the dimensionless energy parameter with $\epsilon_0^{(l)}$ being the resonance energy and $\gamma^{(l)}$ identified with the resonance width. All of the parameters are calculated for a given base pair l.

In the main body of the text, we investigate the influence of the temperature on the transition rates. These results are extracted from the temperature broadening parameter ε, which is a linear function of the temperature. To verify whether the given curve follows a Fano profile, we performed a fit in biologically relevant ϵ–range [9]. The fitting procedure was carried out using nonlinear least squares regression via the Levenberg-Marquardt algorithm, as implemented in the `curve_fit` function from the `Julia LsqFit` library. The results are given in Fig. S3 for each base pair and the parameters of the curves are written in Tab. S2.

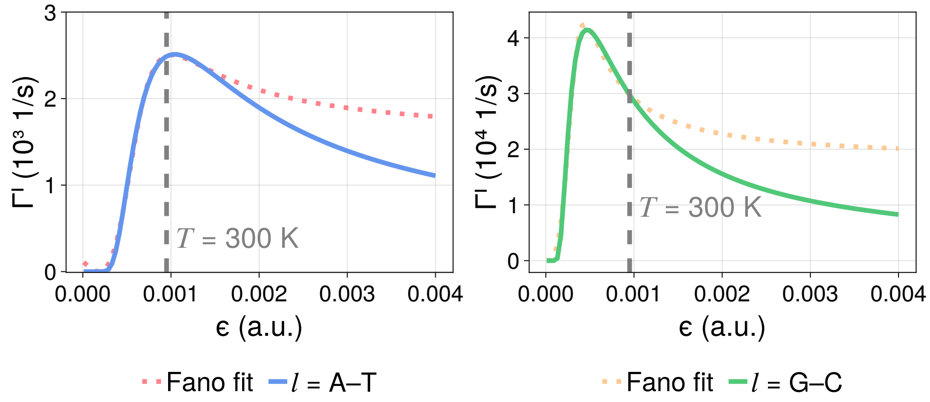

Figure S3: Fano curve fitting (the left panel) and l = G–C (the right panel). The approximation aimed to be the most accurate in the physical domain, with biologically relevant temperatures ranging from 80 to 368 K following the ref. [9].

Table S2: Fano curve parameters for DNA base pairs.

Base pair l	ω [a.u.]	$\Gamma^{(l)}$	$\epsilon_0^{(l)}$	$q^{(l)}$	$\alpha^{(l)}$
A–T	4.003×10^{-3}	0.00081	0.00050	0.795	1535.4995
G–C	2.634×10^{-3}	0.00042	0.00028	1.256	47456.6819

S5 Computational Methods

The theoretical results presented in these studies are supported by numerical calculations. They are directly applied to generate the energy potential profile of a proton, the Wigner distribution function associated with the eigenfunction of the harmonic oscillator, and to calculate the transition rates. Below, we list the appropriate source code fragments written in Python's NumPy and the Julia code that are used in these studies.

```
[a_pert, b_pert, c_pert] = np.polyfit(
    x_bohr_perturbation, func_Godbeer_hartree(x_perturbation), 2
)
[a_left, b_left, c_left] = np.polyfit(x_bohr_left, func_Godbeer_hartree
    (x_bohr_left), 2)
[a_right, b_right, c_right] = np.polyfit(
    x_bohr_right, func_Godbeer_hartree(x_bohr_right), 2
)
%\newpage
```

Listing 1: Key code fragment implementing the harmonic approximation based on a 4th-order polynomial for the energy profile potential of the proton.

```
# Wigner function of harmonic oscillator eigenstate
function WDF_func(n::Int, x::Float64, p::Float64, R::Float64; d::
    Float64=0.0)
    x_shift = x - d
    zeta = 2 * x_shift^2 * R + 2 * p^2 / R
    exponen = exp(-R * x_shift^2 - (1 / R) * p^2)
    Laguerre = eval_genlaguerre(n, zeta)
    return (-1)^n / pi * exponen * Laguerre
end
```

Listing 2: Source of code implementing the exact form of the Wigner distribution function

for the harmonic oscillator eigenfunctions.

```julia
# gaussian approximation to delta
gauss_dirac(delta_E, a) = exp(-delta_E^2 / (2*(a)^2)) / (sqrt(2pi) * (a))

U_barrier(x) = a_pert * x^2 + b_pert * x + c_pert

hbar = 1.0                 # atomic units

# Gamma element computed with Wigner-function overlap
function gamma_element(n_i::Int, n_f::Int, E_i::Float64, E_f::Float64, dL::Float64, dR::Float64,
                       L::Float64, R::Float64, a::Float64;
                       xgrid = range(-10.0, 10.0, length=200), pgrid = range(-16.0, 16.0, length=200))

    xs = collect(xgrid); ps = collect(pgrid)
    delta_x = step(xgrid); delta_p = step(pgrid)

    nx = length(xs); np = length(ps)
    WDF_C = zeros(Float64, nx, np)
    WDF_T = zeros(Float64, nx, np)

    for (i,x) in enumerate(xs), (j,p) in enumerate(ps)
        WDF_C[i,j] = WDF_func(n_i, x, p, L, d=dL)
        WDF_T[i,j] = WDF_func(n_f, x, p, R, d=dR)
    end

    sumC = sum(WDF_C) * delta_x * delta_p
    sumT = sum(WDF_T) * delta_x * delta_p

    integrand_sum = 0.0
    for i in 1:nx, j in 1:np
        integrand_sum += WDF_C[i,j] * WDF_T[i,j] * abs2(U_barrier(xs[i]))
    end
    integral_xp = (2pi * hbar) * integrand_sum * delta_x * delta_p

    delta_E = E_f - E_i
    delta_part = gauss_dirac(delta_E, a)

    return (2pi / hbar) * integral_xp * delta_part
end
```

Listing 3: Key code fragment implementing the partial transition rate $\Gamma_{i \to k}$.

```julia
# Ei, Ef arrays correspond to the same isotope; includes Boltzmann weights over initial states
function compute_rate_curve(Ei::AbstractVector{Float64}, Ef::AbstractVector{Float64},L::Float64, R::Float64, dL::Float64, dR::Float64, a_range::AbstractVector{Float64}; T::Float64 = 300.0, xgrid = range(-10.0, 10.0, length=200), pgrid = range(-16.0, 16.0, length=200))

    kB_au = 3.166811563e-6

    gamma_vals = Float64[]
    for a in a_range
        beta = 1.0 / (a)
        weights = exp.(-beta .* Ei)
        Z = sum(weights)
        p = weights ./ Z
        gamma_au = 0.0
        for (j, E_i) in enumerate(Ei)
            for (k, E_f) in enumerate(Ef)
                n_i = j -1
                n_f = k - 1
                gamma_elem = gamma_element(n_i, n_f, E_i, E_f, dL, dR, L, R, a;
                                        xgrid=xgrid, pgrid=pgrid)
                gamma_au += p[j] * gamma_elem
            end
        end

        # convert atomic-unit rate to s^-1 (1 a.u. time = 2.418884e-17 s)
        rate_s = gamma_au / (2.418884e-17)
        push!(gamma_vals, rate_s)
    end
    return gamma_vals
end
```

Listing 4: Key code fragment implementing the the transition rate Γ with the Boltzmann weights.

References

[1] A. D. Godbeer, J. S. Al-Khalili, and P. D. Stevenson. Modelling proton tunnelling in the adenine-thymine base pair. *Phys. Chem. Chem. Phys.*, 17:13034–13044, 2015.

[2] L. Slocombe, M Sacchi, and J. Al-Khalili. An open quantum systems approach to proton tunnelling in DNA. *Commun. Phys.*, 5:109–118, 2022.

[3] L. Slocombe, M. Winokan, J. Al-Khalili, and M. Sacchi. Proton transfer during dna strand separation as a source of mutagenic guanine-cytosine tautomers. *Commun. Chem.*, 5:144–153, 2022.

[4] C. K. Zachos, D. B. Fairlie, and . T. L. Curtright. *Quantum Mechanics in Phase Space. An Overview with Selected Papers.* World Scientific, 2005.

[5] T. Myint-U and L. Debnath. *Linear Partial Differential Equations for Scientists and Engineers.* Birkhäuser Boston, 2007.

[6] G. B. Arfken, H. J. Weber, and F. E. Harris. *Mathematical Methods for Physicists. A Comprehensive Guide.* Academic Press, seventh edition, 2013.

[7] I. Białynicki-Birula, M. Cieplak, and J. Kamiński. *Theory of Quanta.* Oxford University Press, Inc., 1992.

[8] A. R. P. Rau. Perspectives on the fano resonance formula. *Phys. Scr.*, 69:C10–13, 2004.

[9] E. Marguet and P. Forterre. DNA stability at temperatures typical for hyperthermophiles. *Nuc. Acids Res.*, 22:1681–1686, 1994.
